# Meaning in life: resilience beyond reserve

**DOI:** 10.1186/s13195-018-0381-z

**Published:** 2018-05-24

**Authors:** David Bartrés-Faz, Gabriele Cattaneo, Javier Solana, Josep M. Tormos, Alvaro Pascual-Leone

**Affiliations:** 1 Departament de Medicina, Facultat de Medicina i Ciències de la Salut i Institut de Neurociències, Universitat de Barcelona, Barcelona, Spain; 2 Institut d’Investigacions Biomediques August Pi i Sunyer (IDIBAPS), Barcelona, Spain; 3 Institut Guttmann, Institut Universitari de Neurorehabilitació adscrit a la UAB, Badalona, Spain; 4 Universitat Autònoma de Barcelona, Bellaterra, Spain; 5 Fundació Institut d’Investigació en Ciències de la Salut Germans Trias i Pujol, Badalona, Barcelona, Spain; 6 Berenson-Allen Center for Noninvasive Brain Stimulation, Division of Cognitive Neurology, Beth Israel Deaconess Medical Center, Harvard Medical School, Boston, MA USA

**Keywords:** Cognitive reserve, Meaning in life, Sense of coherence, Purpose in life, Cognition, Affective status

## Abstract

**Background:**

The contribution of psychological factors to brain health and resilience remains poorly investigated. Furthermore, their possible interaction with ‘classical’ cognitive reserve (CR) estimates in predicting perceived mental health and cognitive status has not been specifically addressed.

**Methods:**

We obtained data from 1081 adults responding to questionnaires on the three meaning in life (MiL) dimensions: purpose in life (PiL), sense of coherence (SoC), and engagement with life (EwL). A questionnaire on CR variables was also administered. The outcome measures were self-reported cognitive function and affective status (depression, stress, and anxiety). Multiple linear regression analyses were used to evaluate the association between sociodemographic variables, MiL dimensions, and CR with the two selected outcomes. Mediation analyses, adjusted for age and gender, were applied to determine whether the MiL dimensions mediated the putative effects of CR on self-reported mental and cognitive health.

**Results:**

All three MiL components, but not CR estimates, correlated with the self-reported affective status of the participants. Higher CR, PiL, and SoC (but not EwL) scores significantly correlated with higher perceived cognitive function. Notably, the observed association between the CR measures and self-reported cognitive function was mediated by PiL and SoC.

**Conclusions:**

Psychological MiL dimensions mediate the association between classic CR estimates and self-perceived cognitive function. Further studies on CR could consider including formal measures of such psychological factors to better understand their unique or synergistic contributions, as well as investigate the associated mechanisms maintaining brain function at older ages.

**Electronic supplementary material:**

The online version of this article (10.1186/s13195-018-0381-z) contains supplementary material, which is available to authorized users.

## Background

Largely influenced by the ‘cognitive reserve’ (CR) theory [73, 74], research on factors accounting for interindividual differences in cognitive and brain function in older populations has been dominated by the study of the roles of education, occupation, and social and leisure activities [56]. Recently, with the introduction of broader umbrella terms like ‘lifestyle’ and ‘resilience’, the field has expanded, acknowledging the potential relevance of specific cognitive components such as bilingualism [20], as well as factors like physical activity (e.g., [8]), sleep (e.g., [89]), and diet (e.g., [2]). Particular mental and bodily practices (i.e., meditation; e.g., [22]), and their interactions with both nonmodifiable (e.g., genetic background) and modifiable (e.g., [44]) risk factors, have also been explored. However, psychological factors, including personal resources (e.g., motivational and personality traits, coping strategies or attitude toward life and the future), have received less attention even though they may contribute to late-life health, brain resilience, and the capacity for functional restoration.

The concept of ‘cognitive debt’ was recently proposed, which is related to that of ‘cognitive reserve’, but also considers psychological factors and is associated with maladaptive responses to stress. According to this concept, anxiety, depression, sleep disorders, and repetitive negative thinking represent proxies of ‘cognitive debt’ that deplete resilience to brain diseases with advancing age [48]. Several studies have also reviewed the impact of the psychosocial aspects of life experience on cognitive health in aging, concluding that there is solid evidence linking some personality traits (e.g., neuroticism or conscientiousness) and social connectedness measures (particularly loneliness) with cognitive and clinical measures as well as putative biological mechanisms [83].

In the following sections we briefly summarize available literature around the construct of meaning in life (MiL) and its main three components: purpose in life (PiL), sense of coherence (SoC), and engagement with life (EwL). Our objective was also to investigate the association of these three components with ‘classical’ measures of CR, self-perceived cognitive function, and affective status (depression, anxiety, and stress) in a sample of healthy adults.

### Meaning in life

**Fig. 1:**
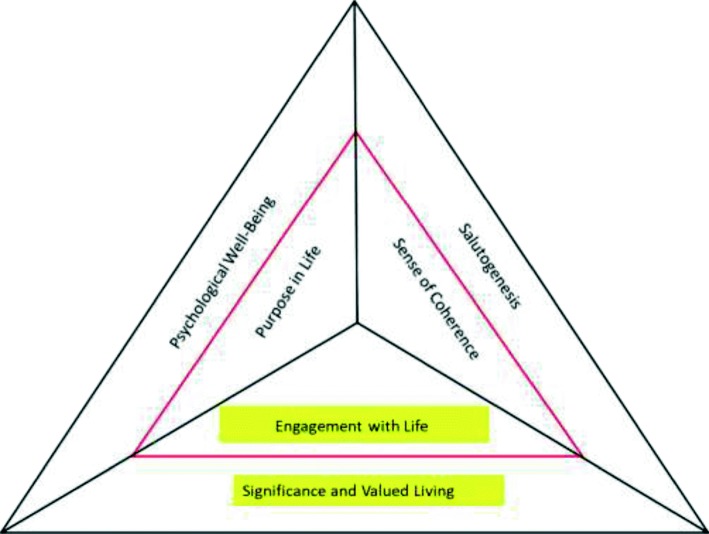
Schematic representation of MiL components (red triangle). Larger black triangle includes theoretical and model perspectives that include these psychological dimensions (see main text)

According to Steger [70], meaning in life is a supraordinate term that encompasses the dimensions of ‘comprehension’ and ‘purpose’. More recently, MiL has been reformulated also referring to the degree of one’s life having ‘value and significance’ [30, 71]. Hence it includes a cognitive component (coherence), a motivational component (goal/purpose), and an affective component (feelings of satisfaction and fulfillment, and that one’s life matters). SoC refers to the ability to ‘make sense, understand one’s life, the external world and how one fits within it’, while PiL encapsulates ‘long-term life aspirations that motivate behavior’. EwL, the affective component, is closely connected to the idea of having significance and life satisfaction and fulfillment (see Fig. 1 and the following main text).

### Purpose in life

The modern origins of the purpose in life (PiL) concept are often linked to Viktor Frankl’s classic texts [25], highlighting the notion that having a strong sense of meaning and future-oriented goals in life (referred to as ‘goal in life’, ‘meaning of life’, or ‘sense in life’ and related to terms such as inner strength, inner or spiritual freedom, and personal value) may result in greater capacity to tolerate challenging and even extreme situations for mental and physical health. According to Carol D. Ryff’s prevalent model [63], PiL is one of the six key components of well-being along with autonomy, personal growth, environmental mastery, positive relationships, and self-acceptance. Regarding the associations of the well-being components with health-related outcomes (see Ryff [65] for a comprehensive review), PiL has been linked to a reduced risk of age-related conditions such as stroke (Kim et al. [35]), disability [36], cardiovascular events, and all-cause mortality [23]. It has also been reported to moderate the impact of biological risk factors (i.e., inflammatory markers; see Ryff et al. [66] for a review). Importantly, PiL has been specifically investigated in the field of cognitive aging and dementia. A higher level of PiL has been associated with better cognitive function in adults without dementia [17] and in middle-aged individuals [42]. Moreover, a seminal longitudinal study [17] found that a high level of PiL predicted a reduced risk for Alzheimer’s disease (AD) or mild cognitive impairment (MCI) after 7 years of follow-up.

### Sense of coherence

SoC is a central component of Antonovsky’s theory of salutogenesis. Aaron Antonovsky was a health sociologist [7] interested in understanding individual differences among those subjected to enduring adverse situations, such as poverty, marginality, immigration, or the Holocaust, particularly why these exposures did not result in poorer health outcomes in some individuals [4]. He defined SoC as a ‘global orientation that expresses the extent to which one has a pervasive, enduring though dynamic feeling of confidence that (1) the stimuli deriving from one’s internal and external environments in the course of living are structured, predictable, and explicable; (2) the resources are available to one to meet the demands posed by these stimuli; and (3) these demands are challenges, worthy of investment and engagement’ [4].

According to the salutogenic model, SoC is considered a universally generalized orientation toward the health end of the health–disease continuum. The salutogenic orientation (vs the pathogenic one) focuses on health promotion and aims to identify ‘salutatory factors’ that actively promote health [6, 78]. SoC represents a cross-cultural construct, allowing individuals to select and use internal and external resources available at their own disposal or in those of others to successfully cope with stressors [4]*.* Such resources are often called generalized resistance resources and include physical and cognitive-emotional capacities, level of education, income, marital status, and community-based facilities. Individuals with a high level of SoC are more likely to adopt flexible strategies to adequately and efficiently use such resources [43].

Epidemiological and clinical studies show that SoC is an important health-promoting resource, strongly linked to mental health and a positive health perception [24]. Along with personality traits, SoC has been positively linked to better health-related quality of life across all age ranges [33]. A strong SoC has been shown to predict subjective well-being to a greater extent than physical disabilities and age itself in older individuals [68], while in very old individuals (> 80 years), SoC has also been associated with better well-being, reduced mortality, a reduced risk of heart failure, and a lower incidence of depression [15, 38, 47]. Moreover, elderly individuals with a high SoC also appear to sustain better cognitive function [62], maintain more of their instrumental activities of daily living [15], and show reduced disability and dependence [80].

### Engagement with life

The third component of the MiL construct refers to the individual’s evaluation of how important, worthwhile, and inherently valuable their life feels and the sense of having a life worth living. Some scholars refer to this as meaning to distinguish it from the purpose dimension (i.e., [26]), whereas others refer to it as significance within the three-component MiL model in which it is associated with the affective component [49]. Previous studies considering the two classical facets of this component (i.e., the presence of meaning and its pursuit or search [72]) have linked this dimension to increased well-being and reduced psychological distress (e.g., [32, 52]). Indeed, this component has been associated with a reduced risk of future depressive symptoms in nonclinical populations (i.e., [50]) and has been reported to buffer the negative effect of depression on posttraumatic stress disorder [58], as well as attenuate or moderate the impact of bullying on suicidal ideation in adolescents (e.g., [31]). However, there is a lack of empirical studies correlating this specific dimension (i.e., separately from purpose and coherence) to cognitive outcomes in older adults or to resilience measures in aging and dementia research. Furthermore, focusing on significant life activities is a central part of therapeutic approaches that promote mental health, such as the acceptance and commitment therapy (ACT) [88]. This approach strongly focuses on the idea of recognizing values meaningful to life that would act as an intrinsic motivating framework and on the concept of engaged or valued living to promote behaviors that are in accordance with life values [29]. Based on these previous studies, the term engagement with life (EwL) was used in the present study to refer to the component covering the relevance of perceived significance and fulfillment of one’s life and the importance of engaging in valued and meaningful activities.

## Methods

### Subjects

The Barcelona Brain Health Initiative (BBHI, www.bbhi.cat) is a prospective longitudinal cohort study aiming to characterize individual protective and risk factors—and the underlying biological mechanisms—for preserved brain function with advancing age. In this investigation, we included 1081 participants (mean (standard deviation) age 52.0 (7.1) years; 680 women) who were aged between 40 and 65 years, had no neurological or psychiatric medical diagnosis, and had provided complete responses to all of the questionnaires. The study was approved by the Comité d’Ètica i Investigació Clínica de la Unió Catalana d’Hospitals.

### Assessments

Participants completed the self-administered questionnaires detailed in the following through the BBHI web-based platform.

The abbreviated version of the Orientation to Life Questionnaire (OLQ-13) was used to estimate SoC. This scale proposed by Antonovsky [5] is widely used and has been validated in the Spanish population [45, 79]. The abbreviated 13-item scale represents a short version of the 29-item original, using a 7-point Likert scale in which a high score corresponds to a high SoC.

The PiL subscale of the Spanish version of Ryff’s Well-Being Scale was used to measure PiL [77] (see Ryff [64] for a review of the other versions). The six-item scale enables the estimation of this component of psychological well-being proposed by Ryff [64]. The Likert scale ranges from 1 (strongly disagree) to 6 (strongly agree), with higher scores corresponding to higher levels of PiL.

The EwL scale [76] was used to assess fulfillment and personal values. This scale is composed of 16 items that are scored on a 5-point Likert scale, ranging from ‘completely disagree’ to ‘completely agree’. Higher scores correspond to higher levels of fulfillment and behavior according to meaningful life values.

Cognitive reserve was estimated using a short questionnaire employed in several neuroimaging-based investigations [14, 16, 69] and subsequently adapted and applied in Spanish samples of healthy elders and AD patients, where it was shown to positively correlate with neuropsychological performance in both samples [61]. The questionnaire used in this investigation obtains information on the classical spheres of CR, and it is structured into five main sections including questions about the attained level of education, occupation, as well as of the extent of engagement in social, leisure, and physical activities. Responses are scored from 0 to 4 points (depending on the question) and the total score out of a maximum of 26 is obtained by directly adding the scores of the responses.

Self-perceived negative affective statuses such as depression*,* anxiety, and stress were evaluated using the subscale of the 21-item version of the Depression, Anxiety and Stress Scales (DASS) [19]. A 4-point Likert scale (1 = never, 4 = always) was used, with higher scores corresponding to a more negative affective state.

To evaluate self-perceived cognitive function*,* we used the Neuro-QoL questionnaire [1, 21]. This instrument includes 12 questions on memory, attention, and reasoning using a 5-point Likert scale (1 = poor/always, 5 = excellent/never). Higher scores correspond to higher levels of self-perceived cognitive function.

### Statistical analyses

Data analyses were performed in three successive steps. The associations between age and gender, the main sociodemographic variables, and the PiL, SoC, and EwL questionnaire scores were analyzed, as well as the relationships between the PiL, SoC, and EwL scores, the CR measures, self-perceived cognitive function, and affective status. First, we conducted correlation and factor analyses, using principal component estimation methods and an oblique oblimin rotation to explore the relationship and overlap among the three dimensions. The latter analysis identified three separate factors closely corresponding to the SoC, PiL, and EwL measures (see later). However, for the purposes of interpretation, all further analyses were undertaken using the original values for each scale, since these have been validated in the Spanish population.

Second, multiple linear regression analyses were undertaken to determine which of the variables (sociodemographic, MiL dimensions, or CR measures) were associated with the two main outcome variables (self-perceived cognitive function and affective status). Finally, on the basis of these results, analyses adjusted for age and gender were performed to determine whether the MiL dimensions mediated the putative effects of CR on self-reported mental and cognitive health.

All statistical analyses were performed using SPSS version 20.0 (Statistical Package for Social Sciences, Chicago, IL, USA). After data were examined for normality, Spearman’s rank correlation was used for the correlation analysis and stepwise linear regression models were run, controlling for multicollinearity. Gender effects (categorized as ‘woman’, ‘man’) were analyzed using univariate ANOVA. Mediation analyses were undertaken using an SPSS macro, the Preacher and Hayes Mediation Procedure [60]. All obtained values were considered significant when *p* < 0.05.

## Results

### Sociodemographic variables and MiL measures

Age was positively associated with the PiL score, although the coefficients indicated a small effect size for this association (ρ = 0.07, *p* = 0.03). SoC (ρ = 0.03, *p* = 0.38) and EwL (ρ = 0.05, *p* = 0.11) scores did not show significant associations with age. Regarding gender, there were no associations with PiL (*F* = 1.62, *p* = 0.20, η^2^ < 0.01) or SoC (*F* = 0.13, *p* = 0.25, η^2^ < 0.01); however, women presented higher EwL scores than men (*F* = 5.63, *p* = 0.02, η^2^ < 0.01; women = 62.0, men = 61.0).

### Relationship between the MiL measures

Confirmatory factor analysis indicated the presence of three different factors corresponding to SoC, PiL, and EwL. Bartlett’s test revealed a significant relationship between the factors (*p* < 0.01). The Kaiser–Meyer–Olkin test confirmed that the data were suitable for factor analysis (KMO= 0.94). Based on the sample size, the acceptable level of factor loading was set at 0.30 [28]. With few exceptions, the three latent factors loaded the items from the different scales as expected. Factor 3 loaded all of the items from the PiL scale, plus two items from the EwL scale and two items from the SoC scale. Factor 1 loaded 13 of the 16 items from the EwL scale and one item from the SOC scale, while Factor 2 loaded 10 of the 13 items from the SoC scale (see Additional file 1 for each subscale item and its corresponding factor loading). Correlation analysis adjusted for age and gender showed large positive associations between PiL and SoC (ρ = 0.54, *p* < 0.01), PiL and EwL (ρ = 0.65, p < 0.01), and SoC and EwL (ρ = 0.45, *p* < 0.01).

### MiL dimensions, perceived cognitive and emotional status, and CR estimates

Table 1 presents the mean scores of the different MiL dimensions, and the perceived cognitive and mental health measures obtained for the whole sample. As shown in Table 2, individuals with higher scores for the three MiL dimensions reported better cognitive function and lower levels of negative affectivity. These associations persisted when partial correlations were adjusted for age and gender. Moreover, there were small to moderate positive correlations between the CR measure and the PiL, SoC, and EwL scores.

**Table 1:** Age of participants and mean scores in meaning of life scales and other administered scales and questionnaires

	Mean (standard deviation)	Range
Age (years)	52.0 (7.1)	40–65
Meaning in life measures		
Purpose in life	30.3 (4.9)	9–36
Sense of coherence	68.4 (9.2)	36–88
Engagement with life	61.7 (6.9)	28–76
Cognitive and mental health measures		
Cognitive reserve	13.7 (3.2)	4–22
Depression Anxiety and Stress Scale	4.3 (2.9)	0–19
Neuro-QoL	52.9 (6.7)	12–60

**Table 2:** Correlations between dimensions of MiL, CR, and measures of self-perceived cognitive functioning and negative affect (DASS scores)

	Self-perceived cognitive functioning	Negative affect (DASS)	Cognitive reserve
Purpose in life	0.21**	−0.32**	0.25**
Sense of coherence	0.24**	−0.40**	0.18**
Engagement with life	0.18**	−0.30**	0.24**
Cognitive reserve	0.14**	−0.12**	–

*CR* cognitive reserve, *DASS* Depression, Anxiety and Stress Scale, *MiL* meaning in life

Reported values are correlation coefficients; **p < 0.01

We subsequently performed stepwise linear regression analyses to determine which of the sociodemographic variables, MiL dimensions, and CR proxies explained most of the variance of cognitive function and affective status. As negative affective states may bias self-reported cognitive function, we included the DASS score as a regressor in the model, with self-perceived cognitive function the dependent variable. As can be seen in Table 3, age, gender, negative affectivity (i.e., the DASS score), CR, PiL, and SoC, but not EwL, all contributed to explaining some of the variance in the self-perceived cognitive function. In this regard, better subjective cognitive status was associated with a lower age, the male gender, and higher PiL, SoC, and EwL scores. The self-perceived negative affective status was associated with lower PiL, SoC, and EwL scores, but not with the CR or any of the sociodemographic variables.

**Table 3:** Linear regression analysis

	Age	Sex	CR	PiL	SoC	EwL	DASS score
Self-perceived cognitive function	− 0.07*	0.12**	0.08*	0.08*	0.13**	0.02	0.14**
Negative affect (DASS)	0.2	0.01	− 0.02	− 0.10*	− 0.31**	− 0.10*	–

Reported values are standardized β coefficients

*CR* cognitive reserve, *DASS* Depression, Anxiety and Stress Scale, *EwL* engagement with life, *PiL* purpose in life, *SoC* Sense of coherence

*p < 0.05; **p < 0.01

**Fig. 2:**
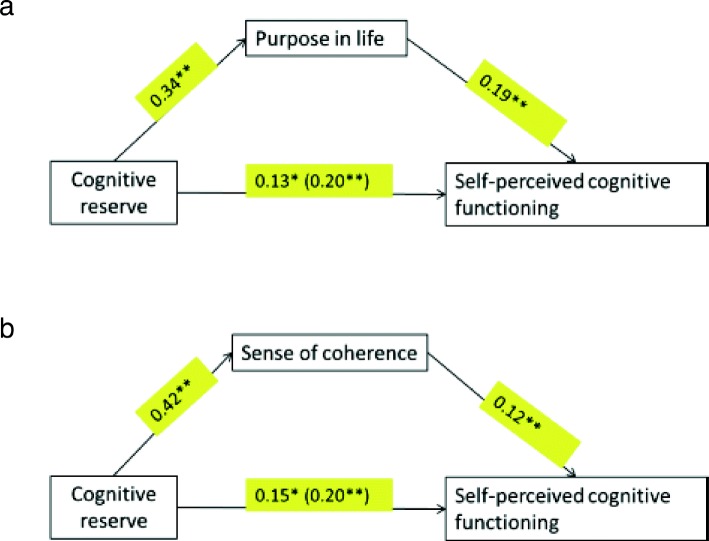
**a** PiL and **b** SoC as mediators of the association between CR and self-perceived cognitive function. Values are *B* coefficients (*p < 0.05; **p < 0.01); values within parentheses represent total relationship between CR and cognitive function when PiL and SoC are not taken into consideration

The putative role of PiL and SoC in mediating the associations between CR and the outcome variables could only be investigated for cognitive function. This was because there was no direct significant association between CR and the self-reported affective status [12]. Bootstrapping, corrected for age and negative affectivity (DASS score), revealed that PiL significantly and partially mediated the association between the proxy measure of CR and self-perceived cognitive function (95% bootstrap CI, 0.03–0.11). Similarly, SoC (95% bootstrap CI, 0.03–0.08) also significantly mediated the association between CR and self-perceived cognitive function (Fig. 2).

## Discussion

The present study, conducted in a relatively large sample of adults, revealed positive associations amongst the MiL dimensions as well as with CR measures. However, not all of the studied variables explained the self-reported health outcomes to a similar degree. Negative affectivity, including depression, anxiety, and stress, only showed an association with the MiL dimensions. By contrast, self-perceived cognitive function also presented an association with CR that was partially mediated by PiL and SoC. In the following sections we discuss these findings in the light of previous literature and highlight its relevance for further studies in the CR field.

### Relationship between the MiL dimensions

Our correlation analyses revealed significant associations amongst the MiL components. Hence, although our confirmatory factor analyses indicated that SoC, PiL, and EwL represented partly independent psychological dimensions, they are strongly interrelated. These positive associations are not surprising given the findings of previous studies. For example, Waytz et al. [81] reported a strong relationship between the presence of meaning, PiL, and satisfaction with life in young individuals. Regarding the engagement and fulfillment dimension, in the original study validating the EwL scale, the authors reported positive associations between this dimension and the six components of the PWB scales including PiL [76]. Furthermore, conceptually, one of the dimensions of SoC, meaningfulness, refers to its motivational component, as it explores the extent to which life makes sense emotionally and whether the demands encountered are worth the commitment and energy so that they are seen as challenges instead of burdens. Hence, this dimension of SoC has elements of both the motivational (purpose) and the affective (fulfillment) aspect of MiL (i.e., [39]). Our observations appear to be in accordance with the conclusions of Martela and Steger [49], who, after reviewing the theoretical and empirical differences and commonalities between the different MiL dimensions, identified separate but synergic and necessary connections in the development of a sense of meaning.

### MiL, CR, and self-reported affective and cognitive outcomes

Regarding the affective status outcome, we observed a direct effect of all three MiL dimensions, with higher scores associated with a better self-perceived mental state. The EwL scale used is composed of two subscales, which reflect the recognition and knowledge of personal values (valued living) and the sense of fulfillment in life as a consequence of recognizing and living in accordance with such values (life fulfillment). In this regard, higher EwL scores correlating with lower perceived negative affectivity are consistent with clinical investigations demonstrating the efficacy of therapeutic approaches that aim to increase value-oriented behaviors in the treatment of anxiety [75]. Similarly, the association of PiL and SoC with affective status is not surprising since general conceptualizations of these two components specifically include the notion that they ‘buffer’ against stressors. Our findings are also in accordance with empirical studies showing a positive direct or moderating effect of these dimensions on mental health outcomes including stress, anxiety, and depression or depressive symptoms (e.g., [24, 84, 85]).

We observed that PiL and SoC positively correlated with self-reported cognitive function. CR was also associated with perceived cognitive function, but this was partially mediated by SoC and PiL. The association between PiL, CR, and cognitive function in our subjects has some parallels in the literature. For example, Lewis et al. [42] reported that PiL scores were positively associated with executive functions, memory, and general cognitive performance across the adult lifespan (32-84 years; see also [84]). Furthermore, McKnight and Kashdan [51] concluded that a certain degree of abstract capacity, insight, and planning explains interindividual differences in purpose formation, such high-order cognitive processes having also been previously associated with CR estimates [57]. Moreover, in addition to the aforementioned studies correlating high PiL with lower incidences of AD and a reduced risk for MCI [17], purpose seems to act through a similar ‘compensatory mechanism’ as CR. For example, PiL has been reported to moderate the relationship between AD pathology and cognition, as participants with higher levels of PiL were observed to show relatively better levels of cognitive performance at higher levels of neuropathology than those with lower levels of PiL [18]. Other studies have indicated that psychological well-being (where PiL is one of the components) may serve as a buffer against adverse health events, specifically interacting with the educational background of the participants, which is a commonly used CR proxy variable. For example, in the Midlife in the United States (MIDUS) survey, Ryff et al. [67] observed that a stable high level of well-being predicted better perceived health, lower incidences of chronic conditions and symptoms, and more preserved instrumental daily activities over time (see also [41, 53, 55]). Notably, the positive effects of maintaining a high level of well-being on health outcomes were especially pronounced in those with a lower level of education.

In accordance with the conceptualization that PiL buffers against stressors, thereby providing an alternative compensatory mechanism to protect cognition, Wilson and Bennett [83] concluded, after reviewing the findings of the Rush Memory and Aging Project, that ‘for the most part, psychosocial measures have not been correlated with neuropathologic changes traditionally associated with dementia in old age’. However, greater levels of PiL have been reported to be associated with fewer subcortical gray matter lacunar infarcts in older adults. This association persisted after adjusting for vascular risk factors, other psychosocial risk factors (i.e., depression, adverse childhood experiences, and loneliness), and physical activity, which have all been previously associated with PiL [86, 87]. Hence, these findings indicate a possible neuroprotective effect of PiL on brain health rather than a compensatory mechanism, a topic of discussion that is currently highly relevant in the CR field (see [9]). It should be noted, however, that the study by Yu et al. [86, 87] did not confirm that a direct protective effect conferred a cognitive advantage in individuals with high PiL as there was no association between the measure (i.e., lacunar infarcts) and a cognitive outcome. Overall, given the previous and current observations of associations between CR and PiL, future studies using biological correlates (e.g., multimodal neuroimaging) should investigate if and how some MiL dimensions contribute to brain resistance beyond the previously reported resilience mechanisms (see a perspective of these terms in [10]).

Associations between SoC and cognitive outcomes and how this may relate to concepts classically linked to CR (‘compensation’) have not been thoroughly investigated. However, a Finnish study [62] reported that SoC was positively associated with both measurable and self-reported cognitive function, with SoC mediating a positive impact of cognitive status on social and mental health (i.e., depression and anxiety). In very old adults, Lövheim et al. [46] observed that decreased cognitive function paralleled a declining SoC over time, while Boeckxstaens et al. [15] reported that a high level of SoC correlated with baseline intact cognition. Moreover, SoC has been found to be associated with education, a common proxy for CR [27], while leisure-time physical activity, another CR proxy, has been reported to have a positive effect on enhancing SoC [54]. In addition, individuals with a high SoC have been shown to exhibit better cognitive performance when encountering an experimental stressor [37]. Altogether, these results agree with our observed associations between SoC and self-reported cognitive status and the involvement of CR in this relationship. None of the aforementioned studies have investigated the biological, in particular cerebral, mechanisms through which high levels of SoC protect cognition in older adults and whether CR moderates this effect. Further longitudinal data from the BBHI study should help to clarify these issues.

Our investigation had several limitations. First, although we included a large sample of participants, the cross-sectional nature of our study meant that the direction of causality of the observed associations could not be determined. Recent longitudinal studies have observed that the stability of the different dimensions of well-being, rather than baseline measurements, correlates with better self-reported [67] and objectively measured [36] health outcomes. Furthermore, our regression analyses might have been limited by the fact that we applied linear regression models to non-normally distributed data. Moreover, we did not include an objective measure (i.e., neuropsychological performance) of cognitive performance and some psychological/psychosocial aspects, such as personality traits or social engagement previously linked to brain health status, were not included in our analyses. However, all of these issues should be addressed when the BBHI study undertakes assessments of the participants, which will include biological characterizations (i.e., neuroimaging and biomarkers). Furthermore, specific questionnaires measuring MiL dimensions other than the ones explored here are available in the literature (e.g., [40, 72]), such as the Meaning in Life Questionnaire [72], which measures purpose and coherence, but not the EwL dimension. Finally, we did not include measures to assess the germane concepts of meaning, particularly that of spirituality, which have been considered in several reports [59].

## Conclusions

In summary, our results suggest that future research on CR, in which cognitive measures are key outcomes, should consider introducing formal ratings and analyzing the impact of MiL components. Some functional neuroimaging data suggest overlapping engagements of particular brain systems and areas for MiL and CR, such as the default-mode network and the anterior cingulate cortex, as well as the dorso and ventrolateral prefrontal regions (reviewed in [3, 13, 66]). Thus, further research is needed to increase our understanding of how MiL and CR components operate through specific or common neural bases to promote brain health. Finally, it should be noted that CR components (cognitive, physical training, and social engagement; reviewed in [11]) and different dimensions of meaning and well-being (e.g., [82, 88]) are amenable to behavioral interventions. Moreover, intervening in one of the components may have an effect on the other dimensions, as has been recently reported in elderly adults in whom physical activity exerted a positive effect on SoC scores [34]. Hence, understanding how such dimensions interact and potentiate one another in response to experimental manipulations could have an impact on the implementation and refinement of future health-promoting multidomain intervention programs.

## Additional file

Additional file 1: Factorial analysis details, normality tests, and graphics of distribution of the scores obtained by participants in the three Meaning in life subcomponent questionnaire. (DOCX 127 kb)
