# Supplementary material for: Meaning in life: resilience beyond reserve
Source: Alzheimers Res Ther. 2018 May 24;10:47. doi: 10.1186/s13195-018-0381-z (PMC5968537; doi:10.1186/s13195-018-0381-z)
Supplement: Supplementary file 1 — Factorial analysis details, normality tests, and graphics of distribution of the scores obtained by participants in the three Meaning in life subcomponent questionnaire. (DOCX 127 kb) [file 13195_2018_381_MOESM1_ESM.docx]

**Additional file**

Table 1. Factor loading of the items included in the three questionnaires measuring each sub-component of Meaning in Life. Only coefficients >0.30 are reported.

Normality tests and graphics of distribution of the scores obtained by participants in the three Meaning in life subcomponent questionnaires:

|  | Kolmogorov-Smirnov | | | Shapiro-Wilk | | |
| --- | --- | --- | --- | --- | --- | --- |
|  | Statistic | gl | Sig. | Statistic | gl | Sig. |
| EwL | ,099 | 1081 | ,000 | ,937 | 1081 | ,000 |
| SoC | ,072 | 1081 | ,000 | ,974 | 1081 | ,000 |
| PiL | ,125 | 1081 | ,000 | ,903 | 1081 | ,000 |


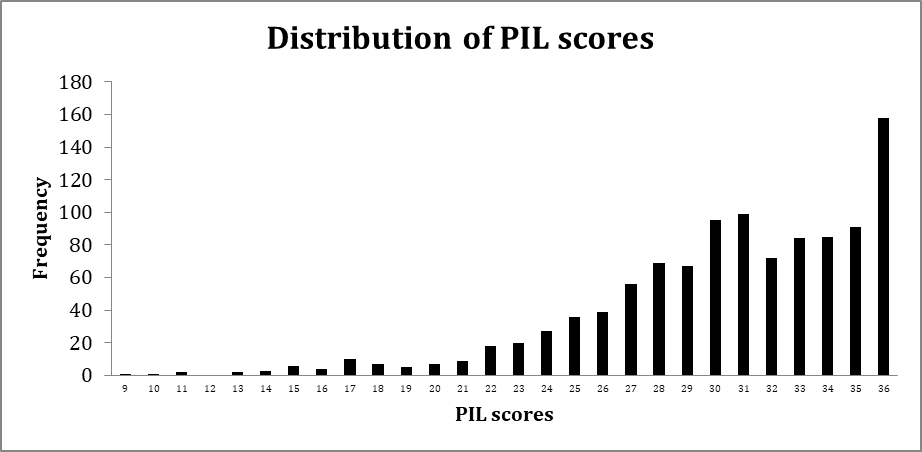


Mean (SD) = 30.3 (4.9)

Range = 9 – 36


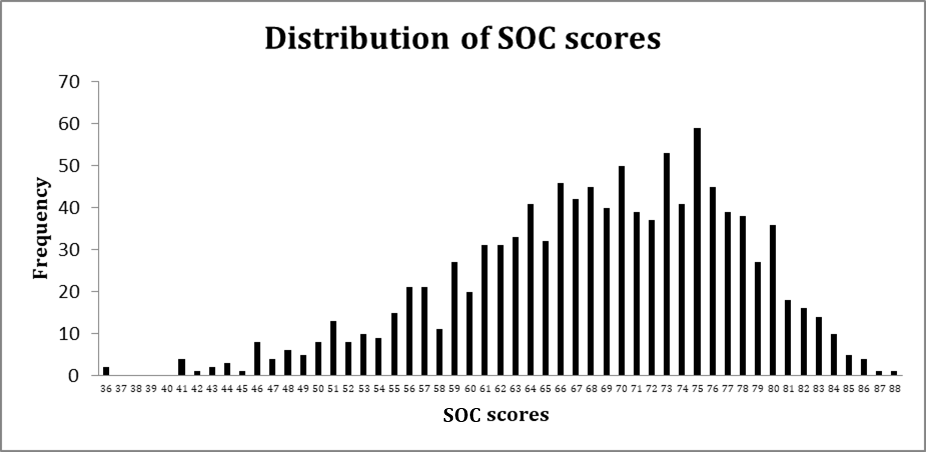


Mean (SD) = 68.4 (9.2)

Range = 36 – 8


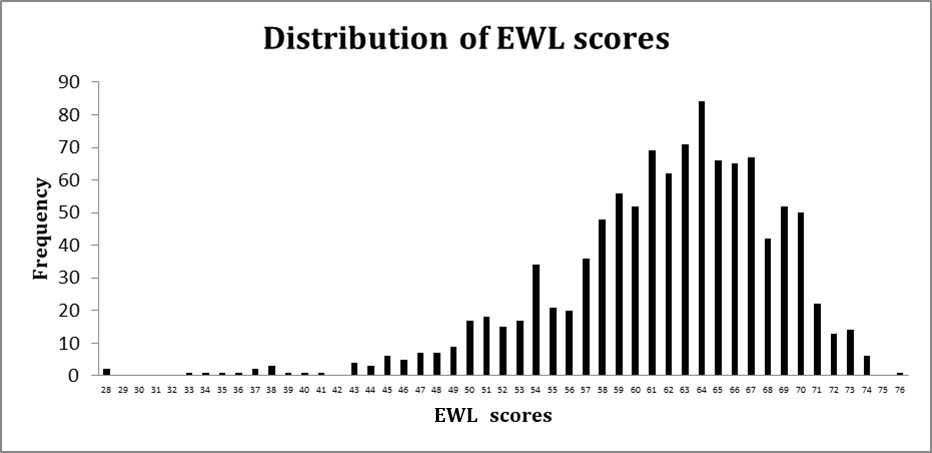
Mean (SD) = 61.5 (6.93)

Range = 28 - 76
